# Supplementary material for: Polystyrene nanoplastics disrupt glucose metabolism and cortisol levels with a possible link to behavioural changes in larval zebrafish
Source: Commun Biol. 2019 Oct 18;2:382. doi: 10.1038/s42003-019-0629-6 (PMC6802380; doi:10.1038/s42003-019-0629-6)
Supplement: Supplementary file 1 — Supplementary Information [file 42003_2019_629_MOESM1_ESM.pdf]

Supplemetary Figures

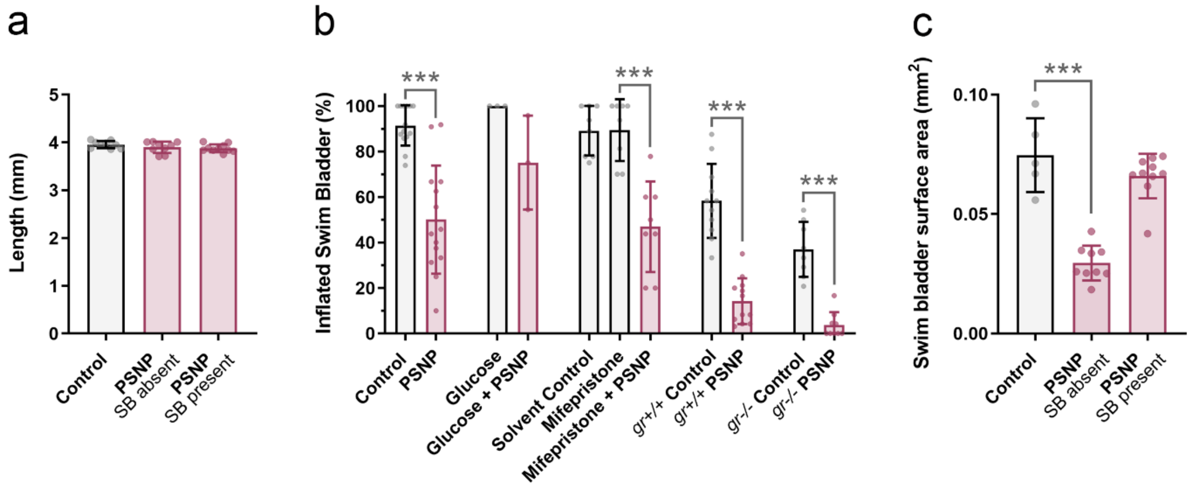

Supplementary Figure 1. Physiological endpoints in zebrafish larvae after PSNP exposure (20 mg L^{-1}) from 72 to 120 hpf. (**a**) Length of wild-type zebrafish larvae at 120 hpf (n = 9-10, each replicate is representing a randomly selected individual larva). (**b**) Percentage of inflated swim bladder in wild-type zebrafish larvae exposed to PSNP in pure egg water, egg water supplemented with 40 mM Glucose or RU486, and in *gr-/-* and *gr+/+* larvae exposed to PSNP in egg water (n = 3-12, with each replicate representing the Inflated Swim Bladder rate in a group of 24 larvae). (**c**) Swim bladder (SB) surface area of zebrafish larvae at 120 hpf with non-inflated SB (SB absent) and inflated swim bladder (SB present). Values are presented as mean ± SD (n = 9-10, each replicate representing a randomly selected individual larva). Asterisks indicate significant differences to controls (*$p < 0.05$, **$p < 0.01$, and ***$p < 0.001$).

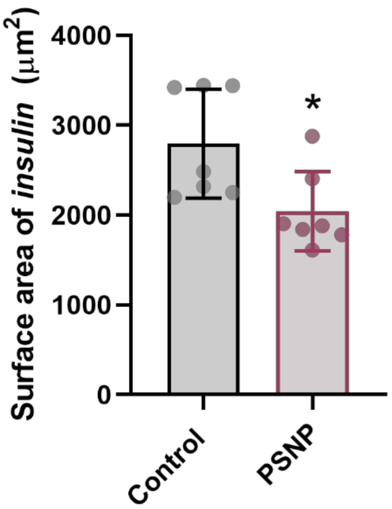

Supplementary Figure 2. Surface area of the insulin-expressing region in 5 dpf zebrafish larvae derived from *in situ* hybridisation. Values are presented as mean ± SD (n = 7 each replicate representing an individual larva). Asterisks indicate significant differences to controls (*$p < 0.05$, **$p < 0.01$, and ***$p < 0.001$).

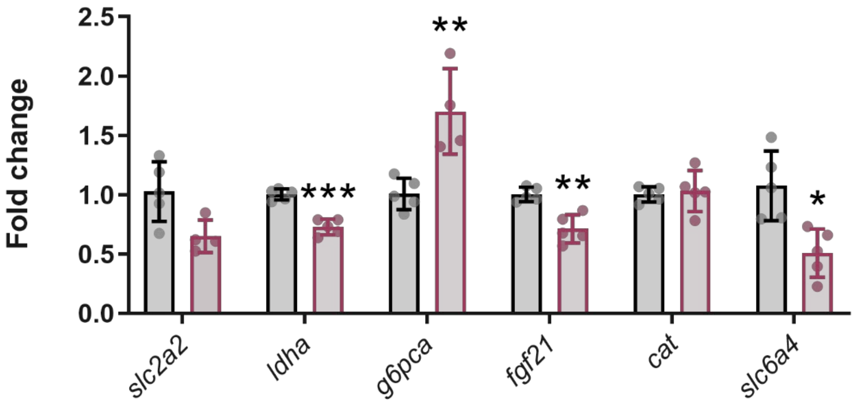

Supplementary Figure 3. Transcriptional changes of genes related to glucose metabolism (*slc2a*, *ldha*, *g6pc*a, *fgf21*), oxidative stress (*cat*), and a membrane protein that transports the neurotransmitter serotonin (*slc6a4*). Values are presented as mean ± SD ($n = 4$-5, each replicate representing a pool of 15 larvae). Asterisks indicate significant differences to controls (*$p < 0.05$, **$p < 0.01$, and ***$p < 0.001$).

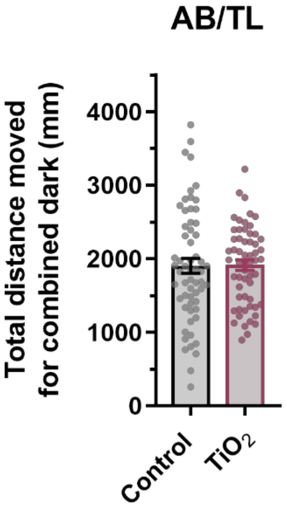

Supplementary Figure 4. Cumulative activity (mm) in the dark phase of TiO_2 exposed zebrafish larvae. TiO_2 nanoparticle (19.5 nm) were in the same size range as PSNP (25 nm) and served as particle control. Data points represent biologically independent replicates from three independent experiments and the error bar indicates the mean ± SEM. TiO_2 exposed zebrafish exhibit an activity pattern alike the control (Control AB/TL: $n = 60$, TiO_2 AB/TL: $n = 57$).

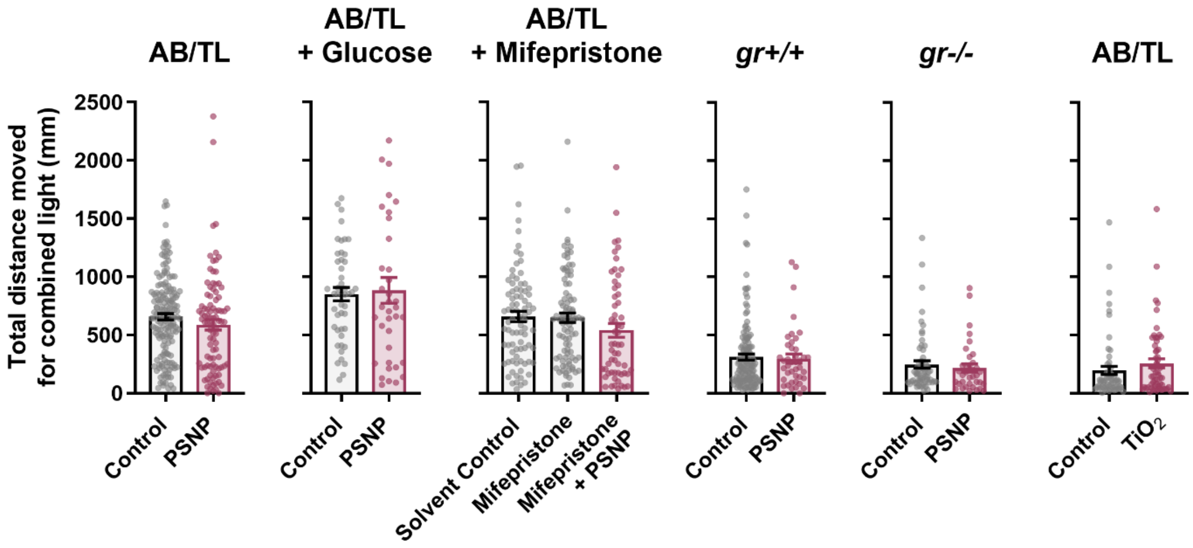

Supplementary Figure 5. Cumulative activity (mm) in all light phases (three times four minutes) tracked for individual larvae. There is no statistical difference between any of the exposure groups and the corresponding control group. Control AB/TL: *n* = 165, PSNP AB/TL: *n* = 91, Control AB/TL + Glucose: *n* = 48, PSNP AB/TL + Glucose: *n* = 38, Control AB/TL + Mifepristone: *n* = 84, PSNP AB/TL + Mifepristone: *n* = 58, Control *gr+/+*: *n* = 136, PSNP *gr+/+*: *n* = 40, Control *gr-/-*: *n* = 63, PSNP *gr-/-*: *n* = 36, Control AB/TL: *n* = 60, TiO_2 AB/TL: *n* = 57). Data points represent biologically independent replicates from at least three independent experiments and the error bar indicates the mean ± SEM.

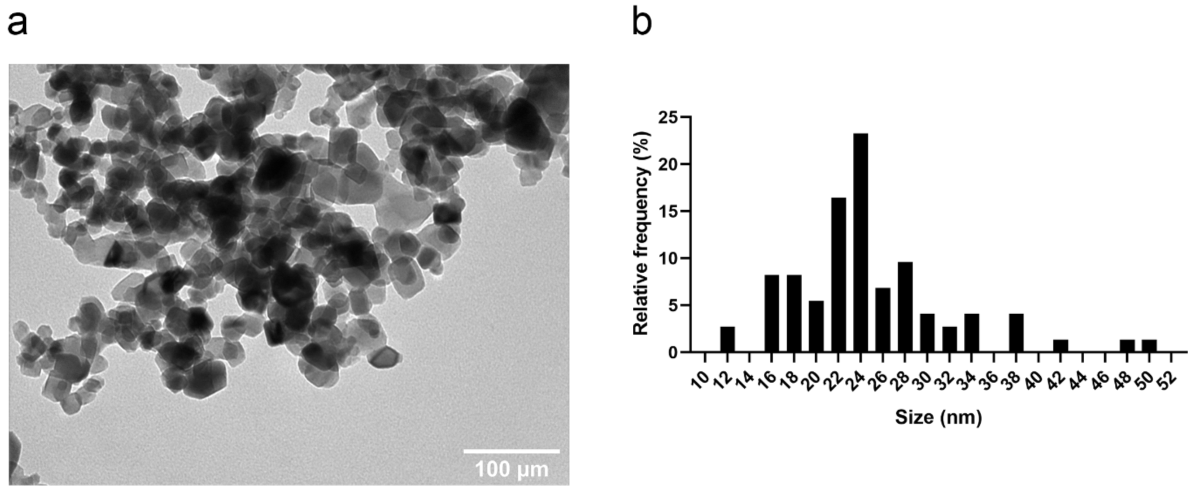

Supplementary Figure 6. Size of TiO_2 nanoparticles used in locomotion assay. (**a**) Transmission Electron Microscopy (TEM) image and (**b**) histogram of TiO_2 nanoparticle diameter in TEM image.

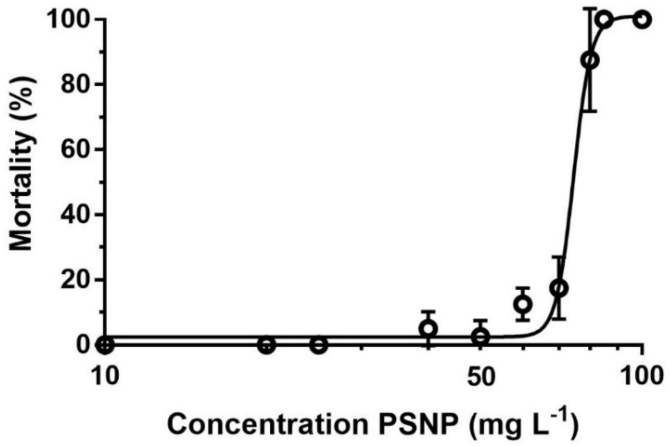

Supplementary Figure 7. Dose-response curve in zebrafish larvae after exposure to PSNP from 72 to 120 hpf. Values are presented as mean ± SD (N = 40, n = 10, each replicate representing the mortality rate in a group of ten larvae).

Supplemetary Table

Supplementary Table 1 Primer sequences used for quantitative Real-Time PCR analysis.

Target gene	Primer Sequence (5' to 3')	Accession no.[a]
rpl13α	Forward: AGC TCA AGA TGG CAA CAC AG	NM_198143.1
	Reverse: AAG TTC TTC TCG TCC TCC GA	
slc2a2	Forward: GGG ATA CAG CTT GGG CGT CAT C	NM_001042721.1
	Reverse: GGA CAA CAT GCC TCC GAC AGA GA	
ldha	Forward: TGG GTC GTT GGA GAA CAT GG	NM_131246.1
	Reverse: CTT GTG GAC GCT CTT CCA GT	
cat	Forward: TAA AGG AGC AGG AGC GTT TGG CTA	NM_130912.2
	Reverse: TTC ACT GCG AAA CCA CGA GGA TCT	
fgf21	Forward: CTC CGT CAA AGG CTC TCC TG	NM_001045324.1
	Reverse: GTG CAG AGT AAT GAT GCT G	
g6pca1	Forward: GAG ACT GGC TGA ACC TCG TC	NM_001003512.2
	Reverse: GAT TGA AAG CAA CGC TGT GA	
slc6a4	Forward: ACATCTCCTCAAAGCCCCAAA	NM_001039972.1
	Reverse: CCACCAGAGTCCTAAATGTTCCA	

[a] GeneBank accession number (http://www.ncbi.nlm.nih.gov).
