# Supplementary material for: Polystyrene nanoplastics disrupt glucose metabolism and cortisol levels with a possible link to behavioural changes in larval zebrafish
Source: Commun Biol. 2019 Oct 18;2:382. doi: 10.1038/s42003-019-0629-6 (PMC6802380; doi:10.1038/s42003-019-0629-6)
Supplement: Supplementary file 2 — descriptions of additional supplementary files [file 42003_2019_629_MOESM2_ESM.pdf]

Description of Additional Supplementary Files

File Name: Supplementary Data 1

Description: The raw data for the generation of graphs in Figure 2, Figure 3, and Figure 4.
