# Supplementary material for: Polystyrene nanoplastics disrupt glucose metabolism and cortisol levels with a possible link to behavioural changes in larval zebrafish
Source: Commun Biol. 2019 Oct 18;2:382. doi: 10.1038/s42003-019-0629-6 (PMC6802380; doi:10.1038/s42003-019-0629-6)
Supplement: Supplementary file 4 — Reporting Summary [file 42003_2019_629_MOESM4_ESM.pdf]

nature research

Corresponding author(s): Nadja R Brun

Last updated by author(s): Jul 9, 2019

Reporting Summary

Nature Research wishes to improve the reproducibility of the work that we publish. This form provides structure for consistency and transparency in reporting. For further information on Nature Research policies, see Authors & Referees and the Editorial Policy Checklist.

Statistics

For all statistical analyses, confirm that the following items are present in the figure legend, table legend, main text, or Methods section.

n/a	Confirmed	
☐	☒	The exact sample size (n) for each experimental group/condition, given as a discrete number and unit of measurement
☐	☒	A statement on whether measurements were taken from distinct samples or whether the same sample was measured repeatedly
☐	☒	The statistical test(s) used AND whether they are one- or two-sided<br>*Only common tests should be described solely by name; describe more complex techniques in the Methods section.*
☐	☒	A description of all covariates tested
☐	☒	A description of any assumptions or corrections, such as tests of normality and adjustment for multiple comparisons
☐	☒	A full description of the statistical parameters including central tendency (e.g. means) or other basic estimates (e.g. regression coefficient) AND variation (e.g. standard deviation) or associated estimates of uncertainty (e.g. confidence intervals)
☐	☒	For null hypothesis testing, the test statistic (e.g. F, t, r) with confidence intervals, effect sizes, degrees of freedom and P value noted<br>*Give P values as exact values whenever suitable.*
☒	☐	For Bayesian analysis, information on the choice of priors and Markov chain Monte Carlo settings
☒	☐	For hierarchical and complex designs, identification of the appropriate level for tests and full reporting of outcomes
☒	☐	Estimates of effect sizes (e.g. Cohen's d, Pearson's r), indicating how they were calculated

Our web collection on statistics for biologists contains articles on many of the points above.

Software and code

Policy information about availability of computer code

Data collection	EthoVision XT® 12, Bio-Rad CFX Manager software, Tecan i-control™
Data analysis	Microsoft Excel, 2016, Graph Pad Prism 8.0, Adobe Photoshop CC 2018

For manuscripts utilizing custom algorithms or software that are central to the research but not yet described in published literature, software must be made available to editors/reviewers. We strongly encourage code deposition in a community repository (e.g. GitHub). See the Nature Research guidelines for submitting code & software for further information.

Data

Policy information about availability of data

All manuscripts must include a data availability statement. This statement should provide the following information, where applicable:

- Accession codes, unique identifiers, or web links for publicly available datasets
- A list of figures that have associated raw data
- A description of any restrictions on data availability

All source data underlying the graphs presented in the main figures are reported in the Source Data File. All data and materials produced by this study are available from the corresponding author upon request.

Field-specific reporting

Please select the one below that is the best fit for your research. If you are not sure, read the appropriate sections before making your selection.

☒ Life sciences ☐ Behavioural & social sciences ☐ Ecological, evolutionary & environmental sciences

Life sciences study design

All studies must disclose on these points even when the disclosure is negative.

Sample size	Sample size was based on experience from previous published experiments leading to significant results.
Data exclusions	No data were excluded.
Replication	Most experiments were replicated at least three times. All replications confirmed the findings.
Randomization	All exposures were always randomized in the multi-well plates.
Blinding	Blinding was not possible as the experiments were conducted by one scientist at a time (but different scientists performed the same experiment at different times) and therefore the samples needed to be known and tracked.

Reporting for specific materials, systems and methods

We require information from authors about some types of materials, experimental systems and methods used in many studies. Here, indicate whether each material, system or method listed is relevant to your study. If you are not sure if a list item applies to your research, read the appropriate section before selecting a response.

Materials & experimental systems

n/a	Involved in the study	
☒	☐	Antibodies
☒	☐	Eukaryotic cell lines
☒	☐	Palaeontology
☐	☒	Animals and other organisms
☒	☐	Human research participants
☒	☐	Clinical data

Methods

n/a	Involved in the study	
☒	☐	ChIP-seq
☒	☐	Flow cytometry
☒	☐	MRI-based neuroimaging

Animals and other organisms

Policy information about studies involving animals; ARRIVE guidelines recommended for reporting animal research

Laboratory animals	Zebrafish strains used for breeding (males and females): AB/TL, Tg(pck1:Luc2,cryaa:mCherry)s95223, grs357
Wild animals	The study did not involve wild animals.
Field-collected samples	The study did not involve samples collected from the field.
Ethics oversight	Zebrafish were handled in compliance with animal welfare regulations and maintained according to standard protocols (http://ZFIN.org). The culture was approved by the local animal welfare committee (DEC) of the University of Leiden and all protocols adhered to the international guidelines specified by EU Animal Protection Directive 2010/63/EU.

Note that full information on the approval of the study protocol must also be provided in the manuscript.
